# Subcutaneous versus retromuscular approach for the minimally invasive surgical treatment of rectus diastasis with concomitant ventral hernia: systematic review and meta-analysis of current techniques

**DOI:** 10.1007/s10029-025-03430-8

**Published:** 2025-08-29

**Authors:** Lidia Castagneto-Gissey, Maria Francesca Russo, Piergaspare Palumbo, James Casella-Mariolo, Vito D’Andrea, Maria Irene Bellini, Giulio Illuminati, Giovanni Casella

**Affiliations:** 1 Department of Surgery, Sapienza University of Rome, Viale Regina Elena, 324, Rome, 00161 Italy; 2 Department of General and Emergency Surgery, Ospedale dei Castelli (NOC), ASL Roma 6, Rome, Italy

**Keywords:** Rectus diastasis, Ventral hernia, Umbilical hernia, Hernia repair, Recurrence, Seroma

## Abstract

**Purpose:**

This systematic review and meta-analysis aims to evaluate the outcomes of laparoendoscopic extraperitoneal techniques for repairing rectus diastasis (RD) with concomitant ventral hernias, focusing on recurrence rates, surgical site occurrences, and the effectiveness of various surgical approaches and mesh placement sites.

**Methods:**

A comprehensive literature search was conducted using PubMed and the Cochrane Library, adhering to PRISMA guidelines. Prospective and retrospective cohort studies involving adults with RD and concomitant ventral hernias were included. Surgical techniques were classified based on working space (subcutaneous vs. retromuscular) and wall repair technique (stapled vs. fascial plication suture). The primary outcome was recurrence of RD or hernia, and secondary outcomes included seroma, surgical site infections (SSIs), and bleeding.

**Results:**

Twenty-two studies comprising 1,616 patients were analyzed. Mean age was 45.6 years, BMI 30.5 kg/m², with a mean follow-up of 10.5 months. All studies were non-randomized and rated as having a “Serious” risk of bias using the ROBINS-I tool. Recurrence occurred in 19 patients (0.99%), with no significant differences between subcutaneous and retromuscular approaches (0.93% vs. 1.16%, *p* = 0.802) or between stapled and fascial plication techniques (0% vs. 1.18%, *p* = 0.090). Seroma rates were significantly higher in the subcutaneous group compared to retromuscular approach (11.8% vs. 0.70%, *p* < 0.001). SSIs were more common in subcutaneous approaches (2.33% vs. 0.58%, 0.005). Bleeding was low across all groups (1.3%), with higher rates in the stapled compared to the fascial plication group (6.39% vs. 0.37%, *p* < 0.001).

**Conclusions:**

Laparoendoscopic extraperitoneal approaches for RD and ventral hernia repair demonstrate favorable outcomes, with low and comparable recurrence rates among subgroups. The subcutaneous approach is associated with a higher risk of seroma formation while the stapled technique may increase bleeding risk. Further studies with higher methodological quality are needed to guide optimal technique selection.

## Introduction

The definition, classification, and management of rectus diastasis (RD) remains complex and a topic of debate, with a range of surgical techniques currently available. To address these shortcomings, the European Hernia Society (EHS) has established a Clinical Practice Guideline for RD management [1]. Nevertheless, precise criteria for defining RD are limited, with RD generally described as an abnormal divarication of the rectus abdominis muscles resulting from thinning and widening of the linea alba greater than 2 cm. Furthermore, the EHS guidelines for the management of RD recommend that, in the absence of new research, it is essential to thoroughly discuss treatment options with patients, ensuring they are well-informed and fully understand the procedures they are consenting to [1].

Over recent decades, minimally invasive surgery has seen significant advancements, fundamentally changing how abdominal wall defects are treated and resulting in reduced pain and better cosmetic outcomes. Managing RD when combined with epigastric and/or umbilical hernias is a debated issue that presents challenges within the abdominal wall reconstruction field. Current guidelines from both the EHS and the American Hernia Society recommend mesh-based repair for RD with concomitant umbilical and epigastric hernias [1]– [2], while for smaller hernias (less than 1 cm), plication of the anterior rectus sheath may suffice [3].

There are currently two main methods for addressing RD alongside ventral hernia repair: either an anterior subcutaneous approach with plication of the anterior rectus sheath or a posterior retromuscular approach [3]. Endoscopic subcutaneous dissection followed by plication of the linea alba with an onlay mesh has emerged as the most commonly reported technique [4]. Each technique offers its own set of benefits and drawbacks, making it practical to classify surgical approaches based on whether they access the abdominal wall from a subcutaneous or retromuscular position relatively to the rectus muscle.

In the late 1990 s, an innovative endoscopic technique for treating ventral hernias with concurrent RD was introduced [5]– [6]. Since then, variations of this technique have been published under different names in various countries, though they maintain the same fundamental surgical concept and mesh placement, with only slight technical modifications.

Presently, no conclusive data exists to guide surgeons in choosing the optimal approach. Rather, each technique is classified by its approach to the abdominal wall, either anterior or posterior to the rectus muscle, although an attempt at classifying and summarizing the characteristics of the main minimally invasive techniques has been made [7].

This systematic review and meta-analysis aims to analyze the outcomes of new laparoendoscopic extraperitoneal techniques with subcutaneous or retromuscular approach for repairing RD with concomitant ventral hernias, highlighting the potential advantages, complications and recurrence rates each technique offers.

## Methods

### Search strategy and selection of trials

A systematic review and metanalysis was carried out according to the Preferred Reporting Items for Systematic Reviews and Meta-Analyses (PRISMA Statement) criteria [8]. This study was registered to the PROSPERO International prospective register of systematic reviews (Registration Number: CRD42025630612). The ROBINS-I tool was used to assess the risk of bias of the studies included in this study [9].

The search for articles was carried out using Pubmed and the Cochrane library. All searches in the electronic databases were carried out through December 2024. The terms of search, extracted from the Medical Subjects Heading (MeSH) were “laparoscopic surgery” OR “minimally invasive surgery” OR “endoscopic surgical procedure” AND “diastasis recti” OR “rectus diastasis” OR “rectus abdominis diastasis” AND “ventral hernia” OR “abdominal hernia” OR “umbilical hernia” OR “epigastric hernia”. Studies were included only if the primary focus of the article was the surgical minimally invasive repair of both rectus diastasis and primary/incisional ventral hernia.

Case reports, editorials, letters, articles not in English were excluded. Studies examining intraperitoneal (i.e. IPOM/IPOM plus) approaches were also excluded.

The PICO strategy was used to formulate the guiding question: ‘In patients undergoing laparoscopic or minimally invasive surgery for rectus diastasis with concomitant ventral hernia (P), how do different surgical techniques (e.g., subcutaneous vs. retromuscular, onlay vs. sublay mesh placement, stapled vs. fascial plication suture,) (I) affect recurrence rates and surgical site occurrences (SSO) (e.g., seroma, bleeding, superficial skin infections) (O)?’ The eligibility criteria for selection of articles, according to the PICO strategy were: prospective or retrospective cohort studies with adults aged > 18 years with diastasis recti and concomitant primary/incisional ventral hernia (population); undergoing minimally invasive surgery for the repair of diastasis recti and ventral hernia (intervention); through different surgical techniques (i.e. stapled vs. fascial plication suture, subcutaneous vs. retromuscular, onlay vs. sublay mesh placement) (comparison); recurrence of RD/ventral hernia, SSOs (seroma, bleeding, surgical site infections) (outcomes).

### Outcome measures

The primary outcome was RD/ventral hernia recurrence. Secondary outcomes included seroma formation, surgical site infections (SSIs), bleeding, and other SSOs such as hematomas and umbilical skin necrosis. Recurrence was defined as a distance between the inner edge of both recti > 20 mm at the supra and infraumbilical level and > 25 mm at umbilical level.

### Data extraction

Two authors (M.F.R. and L.C-G) independently reviewed the title, abstract, and full text of the articles based on the inclusion and exclusion criteria. After the selection process, the following details were independently extracted from each article using a pre-specified data extraction form and entered into a database: first author, publication year, sex, age, BMI, mesh site, main working space, and other outcomes of interest, follow-up duration, recurrence rates, SSOs (seroma, SSI, bleeding and other complications). The surgical techniques were grouped based on factors such as the primary space used for defect repair (subcutaneous or retromuscular), the placement site of the mesh (onlay or sublay), use of endoscopic staplers (stapled or fascial plication suture technique).

### Statistical analysis

Data analyses were carried out using Julius AI [10]. Meta-analysis of proportion was adopted to estimate the prevalence of phenomena of interest. The heterogeneity among the studies was checked using the Cochran’s Q [11] and the I^2^ statistical tests [12]. A Freeman-Tukey transformation [13] was used to calculate the weighted summary proportion under the fixed and random effects model [14]. Publication bias was evaluated through the Begg and Egger tests [15]. Subgroup analysis in terms of SSOs related to surgery, namely retromuscolar vs. subcutaneous, onlay vs. sublay and stapled vs. fascial plication suture techniques was conducted by OpenMeta[Analyst] [16].

## Results

**Fig. 1:**
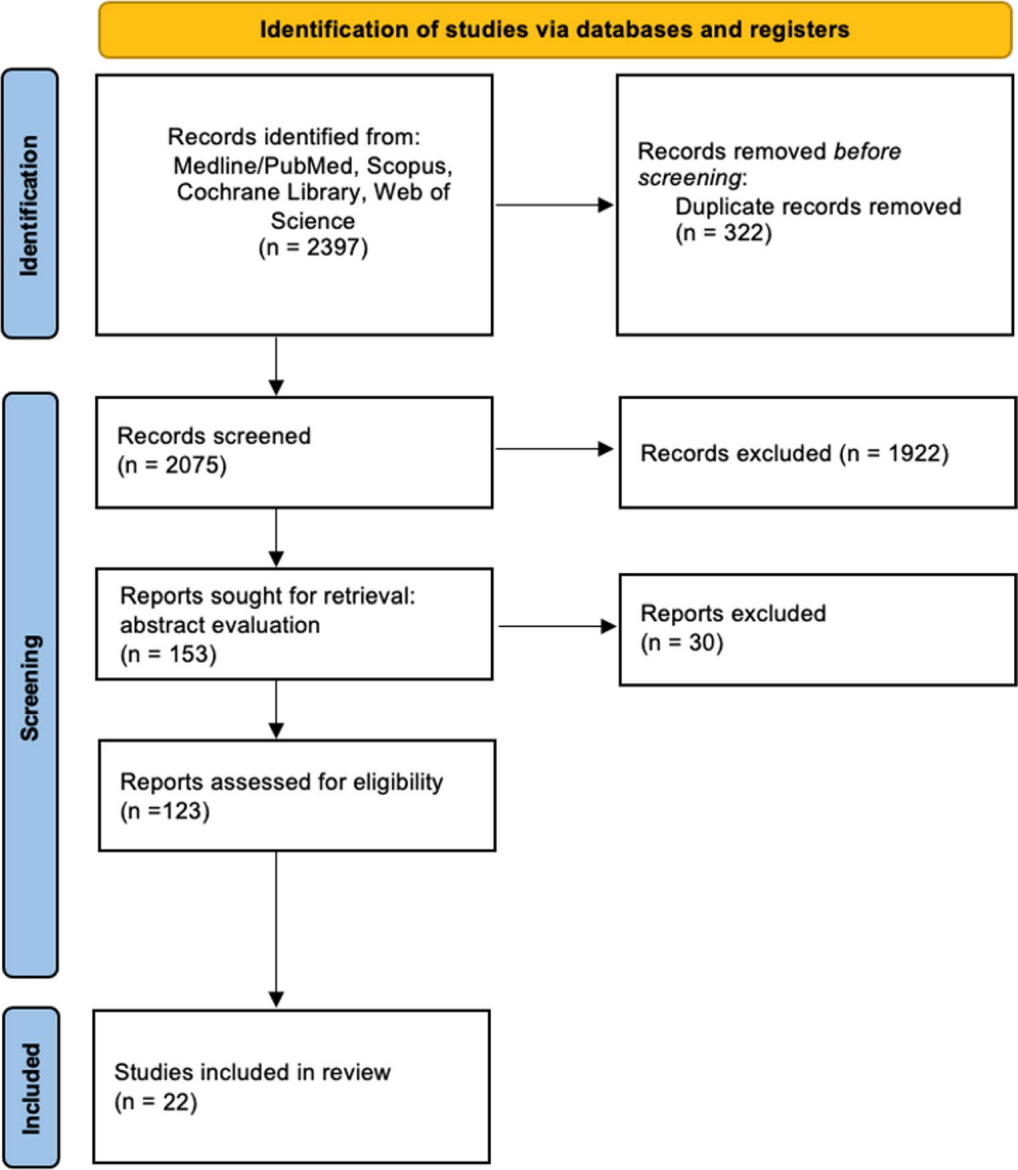
PRISMA 2020 flow diagram for the selection of studies

The literature search retrieved 2397 results, of which 322 were duplicates and excluded from the analysis. One thousand nine hundred twenty-two articles were excluded after title review. The abstracts of the remaining 153 articles were analysed and other 30 studies were excluded. Twenty-two studies were included in the final analysis (Fig. 1).

A total of 22 studies with 1616 patients (613 males, 952 females) were included. Two studies with 25 and 28 patients included, did not specify gender. The studies spanned from 2017 to 2024, with a mean age of 45.6 years, a mean BMI of 30.5 kg/m², and a mean follow-up duration of 10.5 months (Table 1). The studies employed different surgical techniques, with the main working space identified as being either subcutaneous or retromuscular, with mesh placed onlay or sublay.

**Table 1:** Demographic and clinical characteristics of patients in the included studies

Author	Year	Surgical technique	Type of study	*N*° of patients	Male	Female	Age (years)	BMI (kg/m^2^)	Defect size (cm)	Mean Follow-up (months)	Hernia Type
Schwarz et al.	2017	EMILOS	Prospective	25	/	/	53.4	30.3	35.5 cm^2^ (area)	/	Primary & DR
Li et al.	2021	eTPA	Prospective	20	11	9	52.2	28.4	22 cm^2^ (area)	10	Primary & incisional& DR
Reinpold et al.	2018	MILOS	Prospective	615	295	320	60.2	29.7	76.5 cm^2^ (area)	12	Incisional& DR
Manetti et al.	2020	MISAR	Prospective	74	9	65	46.3	24.3	/	6	DR
Carrara et al.	2020	THT	Prospective	110	8	102	43	21.7	16 cm	24	DR
Schwarz et al.	2017	MILOS	Prospective	8	/	/	53.5	25.5	15 cm^2^(area)	1	Primary & DR
Köckerling et al.	2017	ELAR	Prospective	140	90	50	54.7	29.9	59.0	1	Primary, incisional & DR
Kohler et al.	2018	MILAR	Prospective	20	3	17	41	25.3	15.0	5	Primary
Barchi et al.	2018	SVAWD	Prospective	21	12	9	47.5	26.3	74.0	14	Incisional & DR
Claus et al.	2018	SCOLA	Prospective	48	20	28	44.2	27.7	23.0	25	Primary
Dong et al.	2020	SCOLA	Prospective	16	2	14	45.7	29	19.0	/	Primary + DR
Muas et al.	2018	REPA	Prospective	50	3	37	38		/	23	Primary & DR
Cuccomarino et al.	2020	REPA	Prospective	124	6	118	42	22.5	/	12	DR
Signorini et al.	2023	REPA	Retrospective	54	29	25	50.7	28.7	/	6	Primary & DR
Kler et al.	2020	TESLAR	Retrospective	21	8	13	53	29.7	/	/	Primary & DR
Gandhi et al.	2020	EPAR	Prospective	38	14	24	42	28.3	38.0	/	Primary & incisional
Makam et al.	2023	SCOM	Prospective	20	7	13	47	27.9	80.0	14	Primary& DR
Bellido-Luque et al.	2023	FESSA	Prospective	28	28	/	53.4	29.1	37.0	17.3	Primary, incisional & DR
Shinde et al.	2022	SCOLA-modified	Prospective	30	20	10	42.3	28.9	21.0	3	Primary & DR
Valenzuela Alpuche et al.	2024	PeTEP	Retrospective	33	14	19	44.4	> 30	3	4	Primary & DR
Mandujano et al.	2022	R-eTEP	Retrospective	*57*	*20*	37	54.8	32	30 cm^2^(area)	3.38	Primary & incisional
Arias-Espinosa et al..	2024	R-PeTEP	Retrospective	25	23	2	55	30.4	15	1	Primary & DR
Mehta et al.	2024	SCOLA	Prospective	*33*	*12*	21	50	24.28	2.17	18	Primary & DR

### Methodological quality

**Fig. 2:**
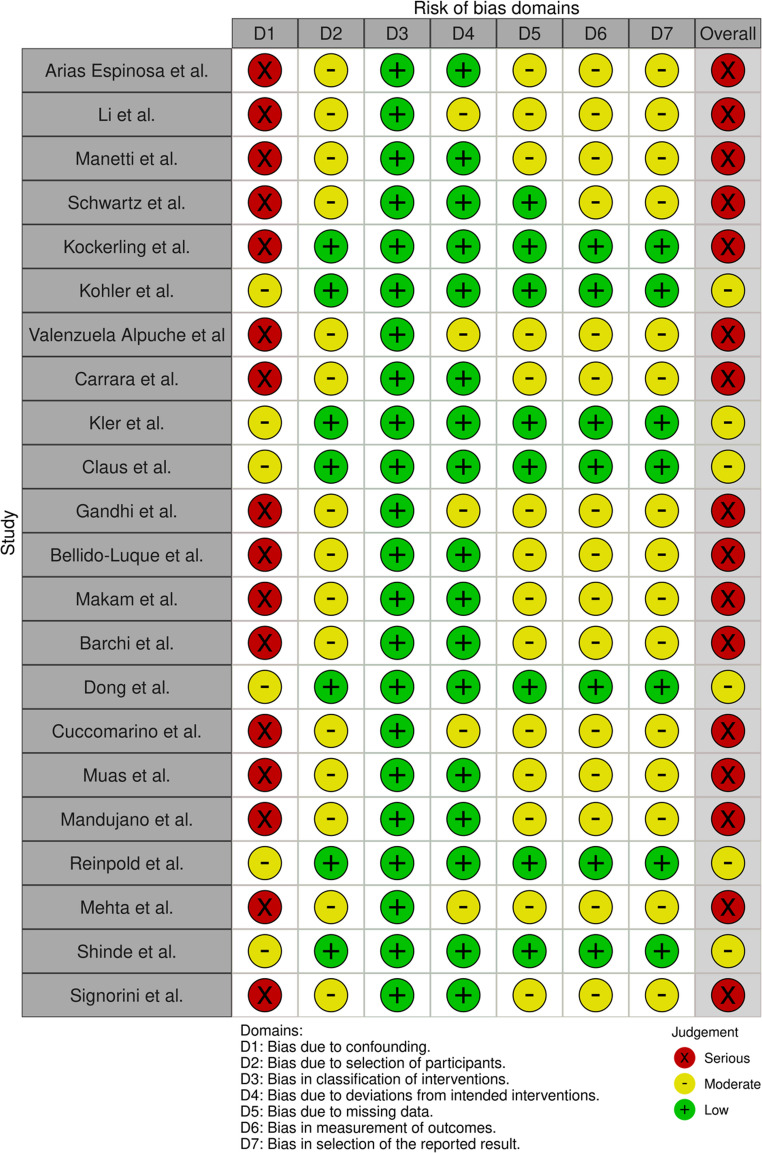
ROBINS-I traffic-light plot

**Fig. 3:**
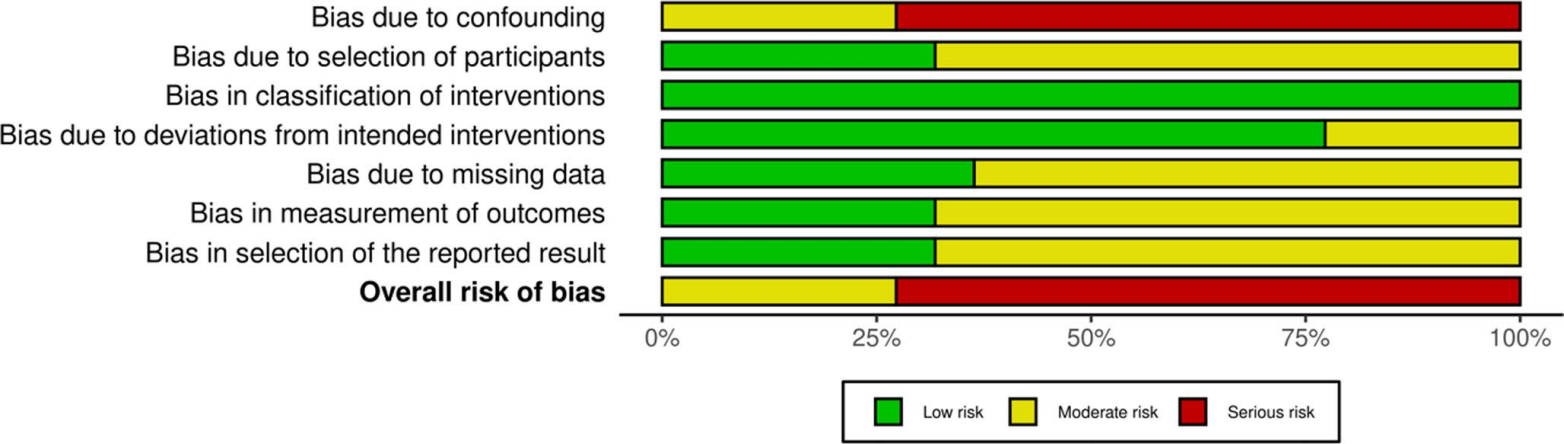
ROBINS-I summary plot

Our systematic application of the ROBINS-I tool revealed that all 22 non-randomized studies included in this review were classified as having a “Serious” overall risk of bias. All studies are retrospective or observational, not randomized controlled trials. According to the ROBINS-I tool, such studies are inherently at higher risk of bias, especially in domains like confounding, selection, and deviations from intended interventions. The studies were published between 2017 and 2024 (Figs. 1, 2 and 3).

### Heterogeneity

Heterogeneity varied across the outcomes analyzed, indicating differences in study populations, surgical techniques, and reporting methods. For seroma, substantial heterogeneity was observed (I² = 89%, *p* < 0.0001), suggesting that factors such as mesh placement technique, surgical approach to the abdominal wall, and patient characteristics, contributed to variability. The highest seroma rates were observed in the subcutaneous approach (16%, 95% CI: 8-28%) which may explain some of the observed heterogeneity.

In contrast, bleeding had significant heterogeneity (I² = 72.2%, *p* = 0.0001), implying considerable variability across studies in reporting this complication. The elevated heterogeneity was largely driven by outlier values, particularly from a subcutaneous study reporting a markedly high bleeding rate (0.64), in contrast to consistently low or zero rates in most other studies. However, in subgroups like the retromuscular and extraperitoneal approaches, heterogeneity was negligible (I² ≈ 0%), underscoring the low risk of bleeding in those anatomical planes.

For SSIs, moderate heterogeneity was observed (I² = 32.6%, *p* = 0.0753), indicating some variation in infection rates across studies. Slight variability may come from differences in antibiotic prophylaxis, wound care protocols, and patient comorbidities. Subgroup analysis revealed very low heterogeneity in retromuscular, extraperitoneal, and extra/preperitoneal approaches (I² = 0%), while the subcutaneous group contributed most of the variability. Although variability was present, it was modest and mostly attributable to the subcutaneous subgroup.

### Primary Outcome

Studies were analysed according to the main working space (subcutaneous vs. retromuscular) and and wall repair technique (stapled vs. fascial plication suture, respectively) (Tables 2 and 3). Not all studies stated a clear definition of recurrence. Most studies diagnosed recurrence based on clinical examination or imaging [22]. A study reported hernia recurrence based on patient self-reporting with selective use of postoperative imaging [19].

**Fig. 4:**
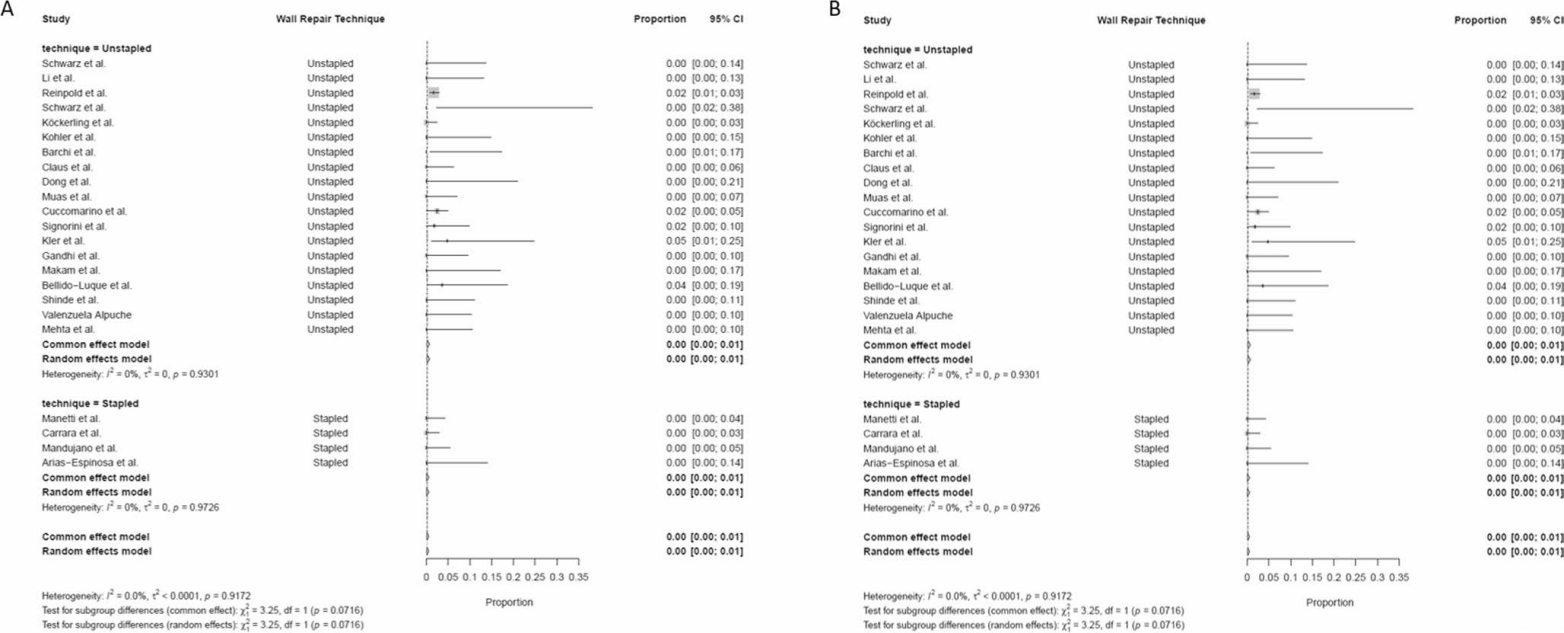
Forest plots illustrating the proportion of recurrence rate across studies subgrouped by subcutaneous vs. retromuscular surgical approach (Panel **A**) and fascial plication suture vs. stapled technique (Panel **B**)

Recurrence was reported in 19 patients (0.99%) across all studies. The comparison between subcutaneous and retromuscular approaches showed no significant difference in recurrence rates (subcutaneous: 0.93%, retromuscular: 1.16%, *p* = 0.802). A higher but not statistically significant recurrence rate was observed in fascial plication suture techniques compared to stapled techniques (1.18% versus 0%, respectively *p* = 0.090) (Table 3) (Fig. 4).

**Table 2:** Surgical technique, approach and mesh placement site

	Main working space	Mesh Site	Wall repair technique
Schwarz et al.	Retromuscular	Sublay	Fascial plication suture
Li et al.	Retromuscular	Sublay	Fascial plication suture
Reinpold et al.	Retromuscular	Sublay	Fascial plication suture
Manetti et al.	Retromuscular	Sublay	Stapled
Carrara et al.	Retromuscular	Sublay	Stapled
Schwarz et al.	Retromuscular	Sublay	Fascial plication suture
Köckerling et al.	Subcutaneous	Onlay	Fascial plication suture
Kohler et al.	Subcutaneous	Onlay	Fascial plication suture
Barchi et al.	Subcutaneous	Onlay	Fascial plication suture
Claus et al.	Subcutaneous	Onlay	Fascial plication suture
Dong et al.	Subcutaneous	Onlay	Fascial plication suture
Muas et al.	Subcutaneous	Onlay	Fascial plication suture
Cuccomarino et al.	Subcutaneous	Onlay	Fascial plication suture
Signorini et al.	Subcutaneous	Onlay	Fascial plication suture
Kler et al.	Subcutaneous	Onlay	Fascial plication suture
Gandhi et al.	Subcutaneous	Onlay	Fascial plication suture
Makam et al.	Subcutaneous	Onlay	Fascial plication suture
Bellido-Luque et al.	Subcutaneous	Onlay	Fascial plication suture
Shinde et al.	Subcutaneous	Onlay	Fascial plication suture
Valenzuela Alpuche et al.	Subcutaneous	Onlay	Fascial plication suture
Mandujano et al.	Preperitoneal	Sublay	Stapled
Arias-Espinosa et al..	Preperitoneal	Sublay	Stapled
Mehta et al.	Subcutaneous	Onlay	Fascial plication suture

**Table 3:** Postoperative SSOs according to subgroups analysis

	Retromuscular (*n* = 858)	Subcutaneous (*n* = 714)	*p* value
Recurrence	10 (1.16%)	6 (0.93%)	0.802
SSI	5 (0.58%)	15 (2.33%)	0.005
Seroma	6 (0.70%)	76 (11.8%)	< 0.001
Bleeding	3 (0.35%)	2 (0.31%)	1.000
Other SSO	5 (0.58%)	10 (1.55%)	0.070
	**Stapled (** ***n*** ** = 271)**	**Fascial plication suture (** ***n*** ** = 1380)**	*p value*
Recurrence	0	16 (1.18%)	0.090
SSI	4 (1.50%)	20 (1.48%)	1.000
Seroma	6 (2.25%)	96 (7.11%)	< 0.001
Bleeding	17 (6.39%)	5 (0.37%)	< 0.001
Other SSO	5 (1.87%)	10 (0.74%)	0.085

### Secondary Outcomes

Several postoperative SSOs were reported across studies. The most common complications included:

- Seroma

**Fig. 5:**
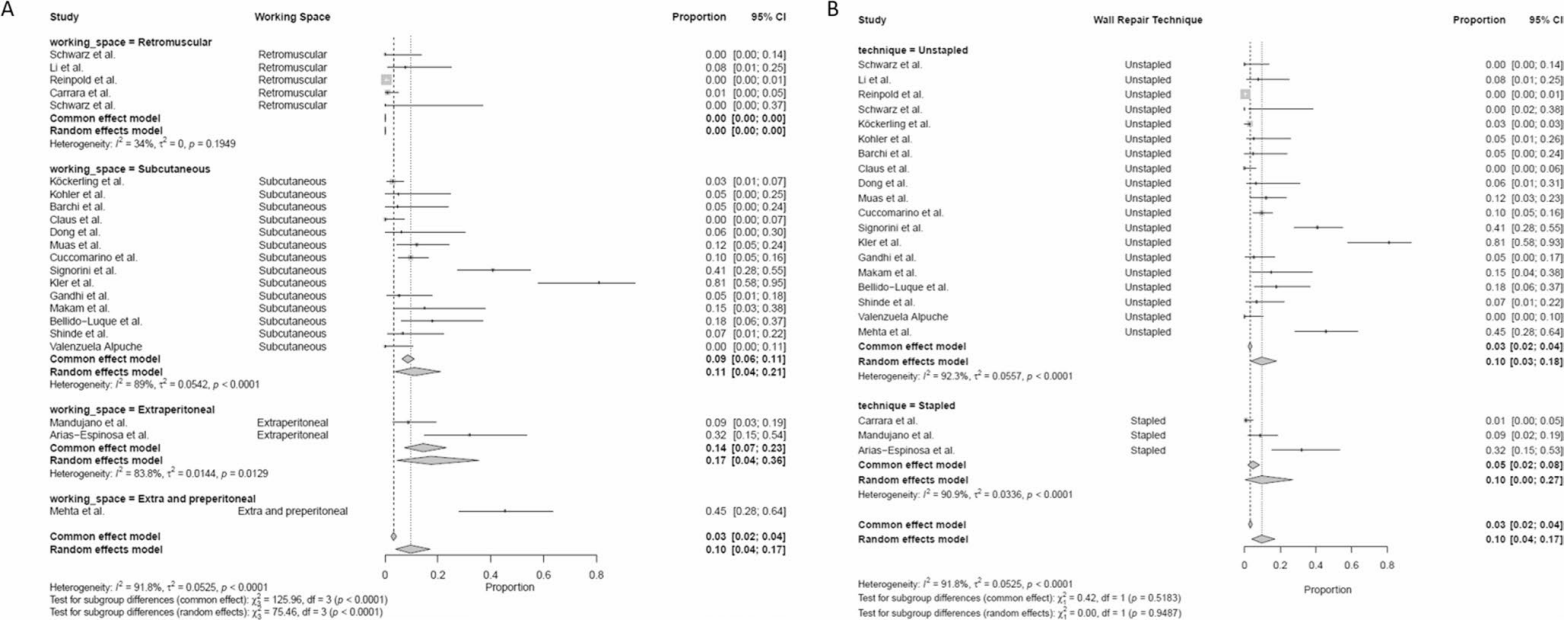
Forest plots illustrating the proportion of seromas across studies subgrouped by subcutaneous vs. retromuscular surgical approach (Panel **B**) and fascial plication suture vs. stapled technique (Panel **B**)

The incidence of seroma varied across different surgical approaches. The overall proportion of seroma was 6.8% (95% CI: 0.7-28%), with significant heterogeneity among studies (I² = 89%, *p* < 0.0001). Regarding the use of stapling methods, the stapled technique had a lower rate of seromas 2.25% compared to fascial plication suture which was associated with an 7.11% seroma rate (*p* < 0.001) (Fig. 5).

- Bleeding

**Fig. 6:**
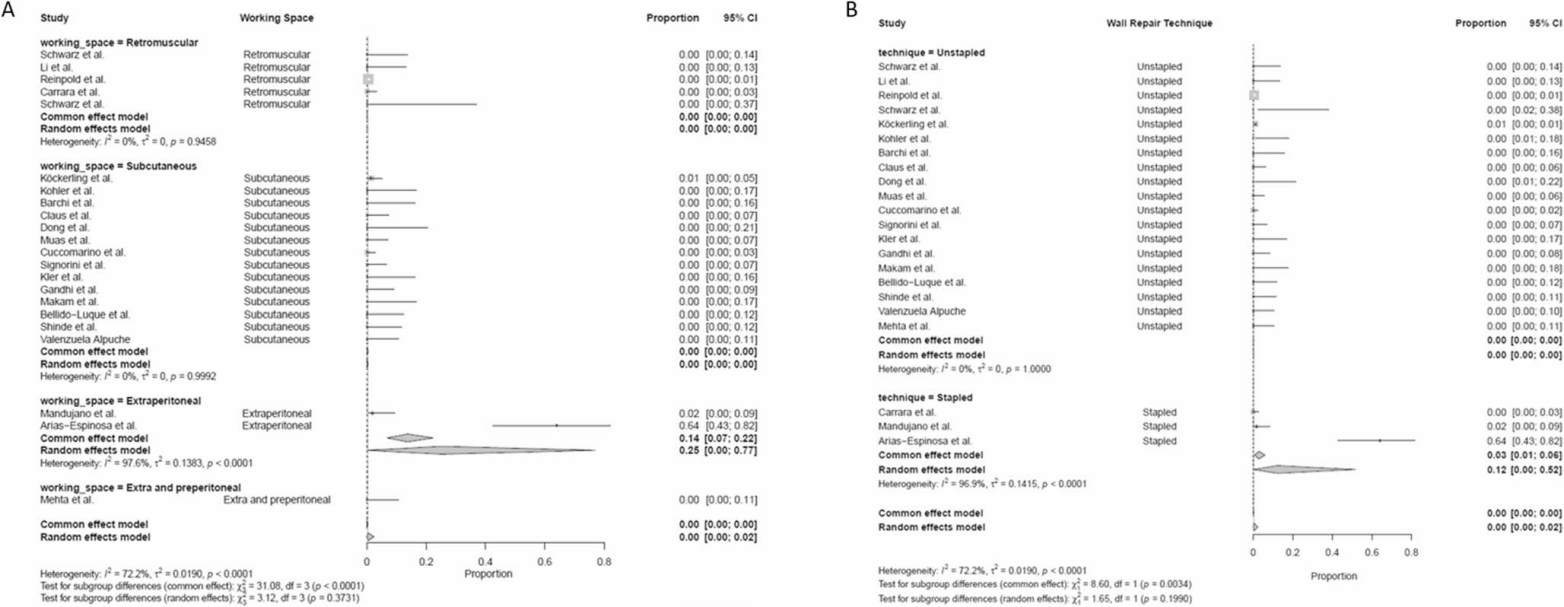
Forest plots illustrating the proportion of bleeding across studies subgrouped by subcutaneous vs. retromuscular surgical approach (Panel A) and fascial plication suture vs. stapled technique (Panel B)

The bleeding rate was low overall at 1.3%. The rates of bleeding in the subcutaneous (0.31%) and retromuscular (0.35%) groups were comparable (*p* = 1.000). The stapled technique had a significantly greater risk of bleeding (6.39%) compared to fascial plication suture techniques (0.37%, *p* < 0.001) (Fig. 6).

- Surgical Site Infections

**Fig. 7:**
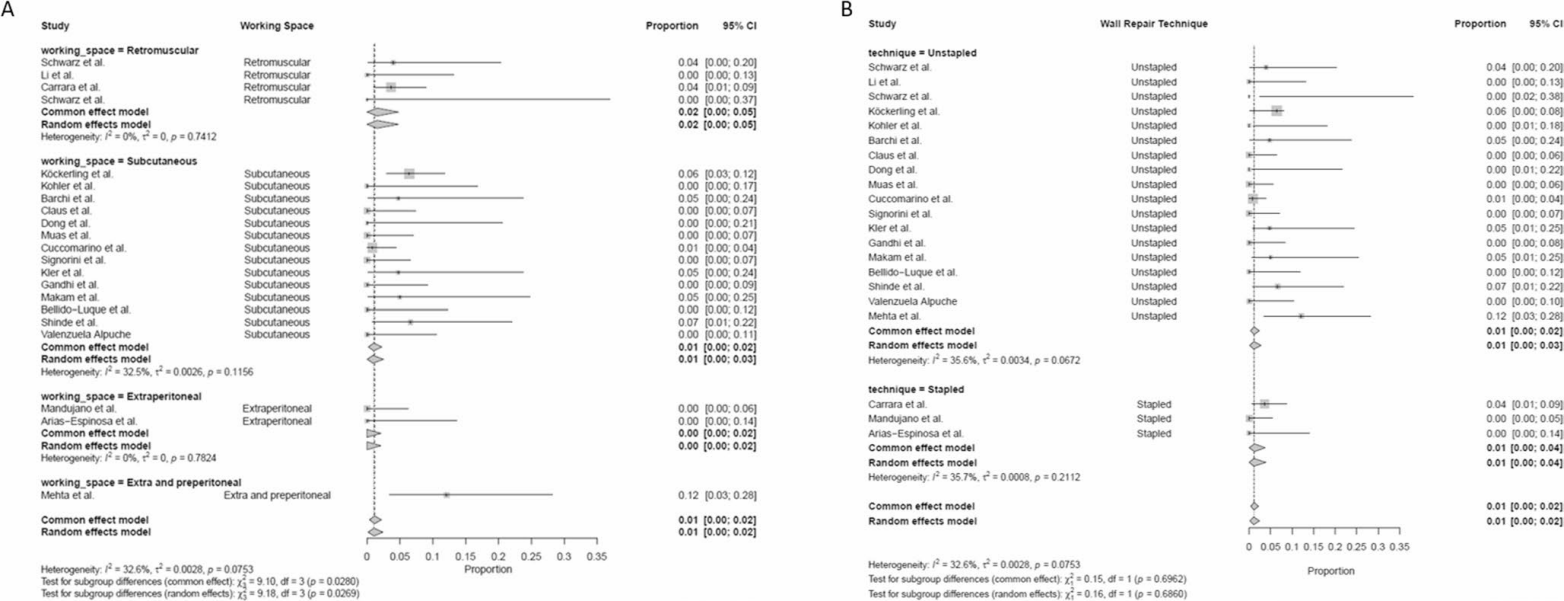
Forest plots illustrating the proportion of SSIs across studies subgrouped by subcutaneous vs. retromuscular surgical approach (Panel A) and fascial plication suture vs. stapled technique (Panel B)

The overall incidence of SSIs was 1.5% (95% CI: 2-5%), with moderate heterogeneity across studies (I² = 32.6%, *p* = 0.0753). The retromuscular approach demonstrated a lower SSI rate of 0.58% (95% CI: 1-5%) compared with the subcutaneous subgroup showing a greater incidence at 2.33% (95% CI: 3-7%), (*p* = 0.005). There were no differences in terms of SSIs between stapled and fascial plication suture techniques (*p* = 1.00) (Fig. 7).

- Other Surgical Site Occurrences

SSOs were reported in 1.03% of subjects overall. Hematomas and umbilical skin necrosis were rare, with hematomas occurring in 0.87% and umbilical skin necrosis in 0.12%. Other minor complications, including wound infections and delayed healing, were reported at low rates across all subgroups. Surgical site occurrences showed some variation across different surgical techniques, though none of the differences reached statistical significance. In retromuscular versus subcutaneous approach, SSOs were reported in 0.58% and 1.55% of cases, respectively (*p* = 0.07). The stapled repair had a higher rate of 1.87% compared to 0.74% in fascial plication suture technique (*p* = 0.085).

### Comparison of surgical techniques

#### Subcutaneous vs. Retromuscular approaches

The comparison between the subcutaneous and retromuscular approaches revealed significant differences in complication rates. Seroma formation was markedly higher in the subcutaneous approach (15.27%) compared to the retromuscular approach (0.70%) (*p* < 0.001). Similarly, SSIs were significantly more frequent in the subcutaneous group (2.66%) than in the retromuscular group (0.58%) (*p* < 0.001). However, recurrence rates (1.16% vs. 1.86%, *p* = 0.81) and bleeding (0.35% vs. 0.28%, *p* = 1.00) were comparable between the two approaches.

#### Fascial plication suture vs. Stapled techniques

The comparison between stapled and fascial plication suture techniques revealed no significant differences in SSIs (1.60% in fascial plication suture vs. 1.47% in stapled, *p* = 1.00), or bleeding (0.36% in fascial plication suture vs. 0% in stapled, *p* = 1.00). However, seroma was more frequent in fascial plication suture techniques compared to techniques where an endoscopic stapler was used (8.45% versus 0.37%; *p* = 0.012). Notably, recurrence was observed only in the fascial plication suture group (1.37%, *p* = 0.05), suggesting a potential advantage of stapling the linea alba in preventing recurrence.

Due to the significant disparity in the number of studies between the two subgroups, with the ‘Stapled’ subgroup consisting of only a single study reporting postoperative SSOs [18], this subgroup analysis lacks the necessary data variability to conduct a valid statistical test. Therefore, comparisons between these subgroups should be considered descriptive rather than conclusive.

## Discussion

This meta-analysis focused exclusively on current minimally invasive approaches for the repair of RD with concomitant ventral hernias, excluding intraperitoneal techniques (e.g., IPOM/IPOM+) and transperitoneal approaches (e.g., ventral TAPP) where RD is not consistently the primary focus [23]. The rationale behind this choice was to highlight the distinctive innovation of extraperitoneal parietoscopic methods, which set them apart from traditional laparoscopic repairs. Moreover, transperitoneal techniques were excluded to maintain greater consistency and homogeneity within the analysis of fully extraperitoneal strategies, thereby highlighting the growing innovation and clinical relevance of minimally invasive parietoscopic approaches that avoid the peritoneal cavity.

A total of 22 articles were examined, covering various surgical techniques often described under numerous names despite sharing similar principles. Differences are primarily technical variations, making standardization difficult due to overlapping features. For simplicity and to improve clarity, this meta-analysis distinguished between subcutaneous (anterior) and retromuscular (posterior) approaches, focusing on the primary working space utilized during the surgical procedure and the site of mesh placement.

The present study indicates that laparoendoscopic extraperitoneal techniques for repairing RD with concomitant ventral hernias are generally effective in terms of recurrence rates and SSOs. A major finding is the low overall recurrence rate of 0.99%, which highlights the efficacy of these minimally invasive techniques. Notably, there was no significant difference in recurrence rates between the subcutaneous and retromuscular approaches, which suggests that both surgical approaches are equally effective in terms of preventing RD and hernia recurrence. Interestingly, a higher but not significant difference was found in terms of recurrence between fascial plication suture of the linea alba compared to the use of endoscopic linear stapling devices (1.18% versus 0%, respectively; *p* = 0.09). Nevertheless, the definition of recurrence varied significantly across studies or was not defined at all, which may have affected the interpretation of results.

Additionally, this study did reveal differences in postoperative SSOs between the surgical approaches. Eight techniques were included in the retromuscular group versus 15 in the subcutaneous one. Seroma formation, a common complication in abdominal wall surgery, was notably higher in the subcutaneous approach compared to the retromuscular approach (11.8% versus 0.70%, *p* < 0.001). This finding aligns with the literature, where the dissection of the subcutaneous plane, although providing easier access, might lead to a greater risk of serum accumulation and subsequent seroma formation compared to the retromuscular approach [24]. Similarly, onlay mesh placement was associated with a higher rate of seroma development compared to the sublay placement (16.01% versus 3.06%, *p* < 0.001). This could also be attributed to the anatomical differences in the way the mesh is positioned, with the onlay mesh being placed subcutaneously rather than beneath the rectus muscles, which may be more prone to causing fluid accumulation in the early postoperative period [25].

The complication rates for SSIs and bleeding were low across all approaches. Significant differences were observed between stapled and fascial plication suture techniques (6.39% vs. 0.37%, *p* < 0.001) while no significant difference was found between subcutaneous and retromuscular dissection with regards to bleeding rates. The minimal rates of bleeding and SSIs are consistent with the general safety of minimally invasive techniques, which are designed to reduce tissue trauma and improve recovery times [26]. Bleeding was the fourth most frequently reported complication after seroma, SSIs and recurrence. Conversely to what has been previously suggested [7], although posterior methods require a dissection mainly occurring in the more vascularized retromuscular space, bleeding was not significantly increased in this group compared to the subcutaneous technique (0.35% and 0.31%, respectively; *p* = 1.00). The stapled division of the linea alba, while efficient for accessing the retrorectus space, may be associated with an increased risk of bleeding. This is likely related to the fact that endoscopic linear staplers are primarily intended for bowel resection and anastomosis, not for abdominal wall dissection—a use that is considered off-label. Applying the stapler in this context may result in suboptimal tissue compression or inappropriate staple height, leading to inadequate hemostasis and a higher incidence of bleeding. On the other hand, SSIs, although relatively low overall, were more commonly encountered in the subcutaneous approach compared to the retromuscular (2.33% versus 0.58%, *p* = 0.005) group.

Two techniques employing staplers for linea alba fascia division, cross-over and stapled plication were analyzed [17]– [18]. While effective for small defects, these methods face challenges in maintaining tension in the posterior plane, particularly for larger hernias. According to a study on an ex vivo model, stapled sutures are capable of withstanding high loads, but their rigidity makes them less deformable compared to handsewn sutures [27]. Authors suggest that this characteristic renders them more appropriate for smaller defects and/or RD, where the anticipated tissue displacement is minimal. In contrast, for larger defects, where tissue displacement is more significant, reinforcing the stapled suture with an oversewing technique enhances its deformability. Hence, stapled plication methods could be proposed for subjects with smaller midline defects and RD but could be less indicated for the treatment of larger ventral hernias.

Fifteen included studies employed an anterior approach with the subcutaneous space serving as the primary operative field. These techniques were previously analyzed in a review exclusively focusing on laparoendoscopic subcutaneous onlay repair and examined the outcomes of 13 studies across 10 countries employing similar surgical methods to address small midline ventral hernias combined with RD [4]. The Authors highlighted the overlapping likenesses of the various surgical terms and proposed merging them under the term ‘Endoscopic Onlay Repair (ENDOR)’. They advocate for this unified term, aiming to foster shared knowledge and improved outcomes in future research.

Since Bellido-Luque’s pivotal 2015 publication [23], the preaponeurotic plane has gained prominence as a suitable area for treating midline defects. Subsequently, other researchers published similar approaches that typically involve subcutaneous dissection, anterior fascia plication with or without onlay mesh placement. These techniques demonstrated low complication rates, despite holding a greater risk of seroma formation (11.8% subcutaneous vs. 0.70% retromuscular approach, *p* < 0.001). This higher incidence is hypothesized to result primarily from subcutaneous dissection and the onlay mesh placement site. Kler et al. [21] reported the highest seroma rate at 81%, potentially due to a higher proportion of biologic mesh use (57%). Seromas were primarily managed conservatively through observation or fine-needle aspiration [4].

While onlay repairs face challenges such as higher rates of seroma, retromuscular mesh placement is often preferred for its lower complication rates. Despite these concerns, anterior approaches provide key advantages, including reduced risk of visceral injury, easier instrument use compared to posterior methods, and complete hernia sac removal, which delivers excellent cosmetic results, particularly for thinner patients. On the other hand, posterior extraperitoneal approaches offer significant advantages, including placement of mesh in an ideal space, namely the retromuscular area, and good functional results with minimal need for drainage. However, these techniques demand expertise due to their steep learning curve and potential complications, such as bulging and hematomas [17].

### Clinical implications

The present findings support the use of laparoendoscopic extraperitoneal techniques for repairing RD with concomitant small-to-moderate size ventral hernias. These techniques offer favorable outcomes with low recurrence rates and minimal postoperative SSOs. However, careful consideration should be given to the increased risk of seroma formation associated with the subcutaneous approach and onlay mesh placement, and augmented bleeding risk with the use of endoscopic staplers as these factors may influence the decision-making process when selecting the optimal technique for individual patients. Surgeon expertise and appropriate case selection remain critical factors, especially as these techniques require a learning curve and often entail advanced laparoscopic or endoscopic skills [3, 7, 20].

### Study limitations and future research

Despite the promising findings, several limitations must be considered. The included studies were observational in nature with retrospective analysis. This introduces potential bias and confounding factors, particularly in the choice of surgical technique and patient selection. Additionally, the lack of detailed data on specific patient factors (e.g., comorbidities, prior abdominal surgery) and long-term outcomes limits the ability to fully assess the impact of the different techniques on perioperative outcomes. The reviewed studies included small patient populations and lacked long-term follow-up, limiting conclusions about recurrence and outcomes. Included studies did not differentiate complication rates based on gender, hence a subgroup analysis with regards to gender influence on the main outcomes was not possible.

Methodological quality of included studies varied, with most being retrospective case series or small prospective cohort studies, limiting the overall strength of evidence and increasing the risk of selection bias. Heterogeneity in surgical techniques, follow-up duration, and outcome reporting further affected comparability. Additionally, many studies lacked standardized definitions for complications such as seroma and recurrence.

The variability in surgical techniques, despite being categorized into clear subgroups, still leaves room for variation in execution. Furthermore, this meta-analysis could not account for the learning curve in the earliest cases, which may have negatively affected outcomes. However, it is reasonable to assume that surgeons developed the necessary skills in an experimental setting before performing surgery on the patients included in each study.

Future studies should aim to include larger, multicenter, randomized controlled trials with longer follow-up periods to confirm these findings, possibly standardizing these approaches and their names. Moreover, further exploration into the long-term outcomes, particularly regarding patient satisfaction with cosmetic results and quality of life, would be beneficial in establishing optimal surgical approaches for RD and ventral hernia repair.

## Conclusions

In conclusion, laparoendoscopic extraperitoneal approaches for the repair of RD with concomitant ventral hernias appear to be safe and effective, with low recurrence rates and a limited incidence of major complications. The subcutaneous approach with an onlay mesh placement was associated with a higher risk of seroma formation, while careful consideration should be given to the augmented bleeding risk linked to the off-label use of endoscopic staplers for linea alba division. Standardizing surgical approaches and conducting larger, multicenter trials would be beneficial for confirming the findings and guiding clinical decision-making.

## References

[1] Hernández-Granados P, Henriksen NA, Berrevoet F et al (2021) European hernia society guidelines on management of rectus diastasis. Br J Surg 108:1189–1191. 10.1093/bjs/znab128 34595502 10.1093/bjs/znab128 PMC10364860

[2] Henriksen NA, Kaufmann R, Simons MP et al (2020) EHS and AHS guidelines for treatment of primary ventral hernias in rare locations or special circumstances. BJS Open 4:342–353. 10.1002/bjs5.50252 32207571 10.1002/bjs5.50252 PMC7093793

[3] Bittner R, Bain K, Bansal VK et al (2019) Update of guidelines for laparoscopic treatment of ventral and incisional abdominal wall hernias (International endohernia society (IEHS)): part B. Surg Endosc 33:3511–3549. 10.1007/s00464-019-06908-6 31292742 10.1007/s00464-019-06908-6 PMC6795640

[4] Malcher F, Lima DL, Lima RNCL et al (2021) Endoscopic onlay repair for ventral hernia and rectus abdominis diastasis repair: why so many different names for the same procedure? Surg Endosc 35:5414–5421. 10.1007/s00464-021-08560-5 34031740 10.1007/s00464-021-08560-5

[5] Corrêa MA (1995) Videoendoscopic subcutaneous techniques for aesthetic and reconstructive plastic surgery. Plast Reconstr Surg 96:446–453. 10.1097/00006534-199508000-00030 7624421 10.1097/00006534-199508000-00030

[6] Champault GG, Catheline JM, Barrat C (1999) Parietoscopic treatment of abdominal wall defects: a report of 15 cases. Hernia 3:15–18. 10.1007/BF01576734

[7] Ferrara F, Fiori F (2024) Laparoendoscopic extraperitoneal surgical techniques for ventral hernias and diastasis recti repair: a systematic review. Hernia 28:2111–2124. 10.1007/s10029-024-03144-3 39312025 10.1007/s10029-024-03144-3 PMC11530491

[8] Page MJ, McKenzie JE, Bossuyt PM et al (2021) The PRISMA 2020 statement: an updated guideline for reporting systematic reviews . BMJ n71. 10.1136/bmj.n71 10.1136/bmj.n71 PMC8005924 33782057

[9] Sterne JA, HernÃ¡n MA, Reeves BC, SavoviÄ‡ J, Berkman ND, Viswanathan M et al (2016) ROBINS-I: a tool for assessing risk of bias in non-randomised studies of interventions BMJ. 355:i4919. 10.1136/bmj.i4919 10.1136/bmj.i4919 PMC5062054 27733354

[10] Caesar Labs, Inc (2024) Julius (April 7 version) [Large language model]

[11] Lau J, Ioannidis JP, Schmid C (1997) Quantitative synthesis in systematic reviews. Ann Intern Med 127:820–826 9382404 10.7326/0003-4819-127-9-199711010-00008

[12] Vos T, Barber RM, Bell B et al (2015) Global, regional, and national incidence, prevalence, and years lived with disability for 301 acute and chronic diseases and injuries in 188 countries, 1990–2013: a systematic analysis for the global burden of disease study 2013. Lancet 386:743–800 26063472 10.1016/S0140-6736(15)60692-4 PMC4561509

[13] Freeman MF, Tukey JW (1950) Transformations related to the angular and the square root. Ann Math Stat 21:607–611

[14] DerSimonian R, Laird N (1986) Meta-analysis in clinical trials. Control Clin Trials 7:177–188 3802833 10.1016/0197-2456(86)90046-2

[15] Higgins JP, Thompson SG (2002) Quantifying heterogeneity in a meta-analysis. Stat Med 21:1539–1558. 10.1002/sim.1186 12111919 10.1002/sim.1186

[16] Wallace BC, Dahabreh IJ, Trikalinos TA et al (2012) Closing the gap between methodologists and end-users: R as a computational back-end. J Stat Softw 49:5

[17] Manetti G, Lolli MG, Belloni E, Nigri G (2021) A new minimally invasive technique for the repair of diastasis recti: a pilot study. Surg Endosc 35:4028–4034. 10.1007/s00464-021-08393-2 33661384 10.1007/s00464-021-08393-2 PMC8195785

[18] Carrara A, Catarci M, Fabris L et al (2021) Prospective observational study of abdominal wall reconstruction with THT technique in primary midline defects with diastasis recti: clinical and functional outcomes in 110 consecutive patients. Surg Endosc 35:5104–5114. 10.1007/s00464-020-07997-4 32964305 10.1007/s00464-020-07997-4

[19] Dong CT, Sreeramoju P, Pechman DM et al (2021) Subcutaneous onlay endoscopic approach (SCOLA) mesh repair for small midline ventral hernias with diastasis recti: an initial US experience. Surg Endosc 35:6449–6454. 10.1007/s00464-020-08134-x 33206243 10.1007/s00464-020-08134-x

[20] Fiori F, Ferrara F, Gobatti D et al (2021) Surgical treatment of diastasis recti: the importance of an overall view of the problem. Hernia 25:871–882. 10.1007/s10029-020-02252-0 32564225 10.1007/s10029-020-02252-0

[21] Kler A, Wilson P (2020) Total endoscopic-assisted linea alba reconstruction (TESLAR) for treatment of umbilical/parumbilical hernia and rectus abdominus diastasis is associated with unacceptable persistent seroma formation: a single centre experience. Hernia 24:1379–1385. 10.1007/s10029-020-02266-8 32691174 10.1007/s10029-020-02266-8

[22] Bellido-Luque J, Gomez-Rosado JC, Bellido-Luque A et al (2023) Severe rectus diastasis with midline hernia associated in males: high recurrence in mid-term follow-up of minimally invasive surgical technique. Hernia 27:335–345. 10.1007/s10029-022-02706-7 36454301 10.1007/s10029-022-02706-7

[23] Bellido Luque J, Bellido Luque A, Valdivia J et al (2015) Totally endoscopic surgery on diastasis recti associated with midline hernias. The advantages of a minimally invasive approach. Prospective cohort study. Hernia 19:493–501. 10.1007/s10029-014-1300-2 25142493 10.1007/s10029-014-1300-2

[24] Claus C, Malcher F, Cavazzola LT (2020) Comment to: TESLAR for treatment of umbilical/paraumbilical hernia and rectus abdominus diastasis is associated with unacceptable persistent seroma formation. Should subcutaneous endoscopic mesh placement be abandoned? Hernia 24:1411–1412. 10.1007/s10029-020-02285-5 32840704 10.1007/s10029-020-02285-5

[25] Deshpande GA, Tirpude B, Bhanarkar H et al (2024) Prospective, observational study of intraperitoneal onlay mesh repair with defect closure versus SCOLA for primary ventral hernia. J Minim Access Surg 20:397–402. 10.4103/jmas.jmas_223_23 39730131 10.4103/jmas.jmas_223_23 PMC11601959

[26] Forester E, Sadiq A (2023) Comparative analysis of the efficacy and functionality of abdominoplasty versus minimally invasive techniques in the surgical treatment of diastasis rectus abdominis in postpartum women. Surg Endosc 37:9052–9061. 10.1007/s00464-023-10540-w 37950027 10.1007/s00464-023-10540-w

[27] Lauro E, Corridori I, Luciani L et al (2022) Stapled fascial suture: ex vivo modeling and clinical implications. Surg Endosc 36:8797–8806. 10.1007/s00464-022-09304-9 35578046 10.1007/s00464-022-09304-9

[28] Schwarz J, Reinpold W, Bittner R (2017) Endoscopic mini/less open sublay technique (EMILOS)—a new technique for ventral hernia repair. Langenbecks Arch Surg 402:173–180. 10.1007/s00423-016-1522-0 27766419 10.1007/s00423-016-1522-0

[29] Li B, Qin C, Bittner R (2020) Totally endoscopic sublay (TES) repair for midline ventral hernia: surgical technique and preliminary results. Surg Endosc 34:1543–1550. 10.1007/s00464-018-6568- 30374792 10.1007/s00464-018-6568-3

[30] Reinpold W, Schröder M, Berger C et al (2019) Mini- or less-open sublay operation (MILOS): a new minimally invasive technique for the extraperitoneal mesh repair of incisional hernias. Ann Surg 269:748–755. 10.1097/SLA.0000000000002661 29342018 10.1097/SLA.0000000000002661

[31] Li B, Qin C, Bittner R (2020) Endoscopic totally extraperitoneal approach (TEA) technique for primary ventral hernia repair. Surg Endosc 34:3734–3741. 10.1007/s00464-020-07575-8 32342218 10.1007/s00464-020-07575-8 PMC7326894

[32] Li B, Qin C, Liu D et al (2021) Subxiphoid top-down endoscopic totally preperitoneal approach (eTPA) for midline ventral hernia repair. Langenbecks Arch Surg 406:2125–2132. 10.1007/s00423-021-02259-w 34297175 10.1007/s00423-021-02259-w

[33] Wang T, Tang R, Meng X et al (2022) Comparative review of outcomes: single-incision laparoscopic total extra-peritoneal sublay (SIL-TES) mesh repair versus laparoscopic intraperitoneal onlay mesh (IPOM) repair for ventral hernia. Updates Surg 74:1117–1127. 10.1007/s13304-022-01288-4 35426604 10.1007/s13304-022-01288-4 PMC9213286

[34] Nakabayashi R, Matsubara T, Shimada G (2023) The endoscopic-assisted or endoscopic mini- or less-open preperitoneal (E/MILOP) approach for primary and incisional ventral hernia repair. Asian J Endosc Surg 16:482–488. 10.1111/ases.13206 37218608 10.1111/ases.13206

[35] De-Carvalho JPV, Pivetta LGA, de Freitas Amaral PH et al (2023) Endoscopic mini-or less-open sublay operation (E/MILOS) in ventral hernia repair: a minimally invasive alternative technique. Rev Col Bras Cir 50 10.1590/0100-6991e-20233405-en PMC10595045 36995832

[36] Köckerling F, Botsinis MD, Rohde C et al (2017) Endoscopic-assisted linea Alba reconstruction: new technique for treatment of symptomatic umbilical, trocar, and/or epigastric hernias with concomitant rectus abdominis diastasis. Eur Surg - Acta Chir Austriaca 49:71–75. 10.1007/s10353-017-0473-1 10.1007/s10353-017-0473-1 PMC5368206 28408920

[37] Köhler G, Fischer I, Kaltenböck R, Schrittwieser R (2018) Minimal invasive linea alba reconstruction for the treatment of umbilical and epigastric hernias with coexisting rectus abdominis diastasis. J Laparoendosc Adv Surg Tech 28:1223–1228. 10.1089/lap.2018.0018 10.1089/lap.2018.0018 29620963

[38] Barchi LC, Franciss MY, Zilberstein B (2019) Subcutaneous videosurgery for abdominal wall defects: a prospective observational study. J Laparoendosc Adv Surg Tech 29:523–530. 10.1089/lap.2018.0697 10.1089/lap.2018.0697 30596545

[39] Claus CMP, Malcher F, Cavazzola LT et al (2018) Subcutaneous onlay laparoscopic approach (SCOLA) for ventral hernia and rectus abdominis diastasis repair: technical description and initial results. ABCD. Arquivos Brasileiros de Cirurgia Digestiva (São Paulo) 31:e1399. 10.1590/0102-672020180001e1399 10.1590/0102-672020180001e1399 PMC6284377 30539974

[40] Fiori F, Ferrara F, Gentile D et al (2019) Totally endoscopic sublay anterior repair for ventral and incisional hernias. J Laparoendoscopic Adv Surg Techniques 29:614–620. 10.1089/lap.2018.0807 10.1089/lap.2018.0807 30807248

[41] Juárez Muas DM (2019) Preaponeurotic endoscopic repair (REPA) of diastasis recti associated or not to midline hernias. Surg Endosc 33:1777–1782. 10.1007/s00464-018-6450-3 30229321 10.1007/s00464-018-6450-3

[42] Cuccomarino S, Bonomo LD, Aprà F, Toscano A, Jannaci A (2022) Preaponeurotic endoscopic repair (REPA) of diastasis recti: a single surgeon’s experience. Surg Endosc 36(2):1302–1309. 10.1007/s00464-021-08405-1 33661382 10.1007/s00464-021-08405-1

[43] Signorini FJ, Chamorro ML, Soria MB et al (2023) Preaponeurotic endoscopic repair (REPA) indication in men could be controversial. Hernia 27:431–438. 10.1007/s10029-022-02716-5 36472758 10.1007/s10029-022-02716-5

[44] Gandhi JA, Shinde P, Kothari B et al (2020) Endoscopic preaponeurotic repair (EPAR) technique with meshplasty for treatment of ventral hernia and rectus abdominis diastasis. Indian J Surg. 10.1007/s12262-020-02189-9

[45] Makam R, Chamany T, Nagur B et al (2023) Laparoscopic subcutaneous onlay mesh repair for ventral hernia: our early experience. J Minim Access Surg 19:223–226. 10.4103/jmas.jmas_225_22 37056088 10.4103/jmas.jmas_225_22 PMC10246643

[46] Shinde PH, Chakravarthy V, Karvande R et al (2022) A novel modification of subcutaneous onlay endoscopic repair of midline ventral hernias with diastasis recti: an Indian experience. Cureus 14:1–9. 10.7759/cureus.26004 10.7759/cureus.26004 PMC9288225 35859952

[47] Tsimoyiannis EC, Tassis A, Glantzounis G et al (1998) Laparoscopic intraperitoneal onlay mesh repair of incisional hernia. Surg Laparosc Endosc 8:360–362 PMID: 9799145 9799145

[48] Mehta K, Parmar GVR (2024) An emerging, less explored subcutaneous onlay laparoscopic approach for ventral hernias with concomitant diastasis recti. Sci Rep 14:26938. 10.1038/s41598-024-78398-z 39506083 10.1038/s41598-024-78398-z PMC11541576

[49] Mandujano CC, Lima DL, Xia J, Sreeramoju P, Malcher F (2022) An algorithmic approach for the MIS repair of ventral midline hernias associated with diastasis of the rectus abdominis muscle. J Abdom Wall Surg 1:10864 38314159 10.3389/jaws.2022.10864 PMC10831646

[50] Arias-Espinosa L, Claus CM, Malcher F, Valenzuela Alpuche HA (2024) Robotic preperitoneal extended totally extraperitoneal (R-PeTEP) technique description for ventral hernia repair: preliminary results. Updates Surg 76(7):2715–2722. 10.1007/s13304-024-02002-2 39297928 10.1007/s13304-024-02002-2

[51] Alpuche HAV, Torres FR, González JPS (2024) Early results of eTEP access surgery with preperitoneal repair of primary midline ventral hernias and diastasis recti. A 33 patient case series of PeTEP. Surg Endosc 38(6):3204–3211. 10.1007/s00464-024-10832-9 38637338 10.1007/s00464-024-10832-9

